# Microbiome driven modulation of neurotransmitters: implications for neurotransmission and mood disorders

**DOI:** 10.3389/fmicb.2026.1750377

**Published:** 2026-04-28

**Authors:** Bhagavathi Sundaram Sivamaruthi, Periyanaina Kesika, Chaiyavat Chaiyasut, Durairaj Ragu Varman

**Affiliations:** 1 Office of Research Administration, Chiang Mai University, Chiang Mai, Thailand; 2 Innovation Center for Holistic Health, Nutraceuticals, and Cosmeceuticals, Faculty of Pharmacy, Chiang Mai University, Chiang Mai, Thailand; 3 School of Biomedical Sciences, Sri Balaji Vidyapeeth (Deemed to be University), Puducherry, India

**Keywords:** dopamine, glutamate, microbiota-gut-brain axis, mood disorders, neurotransmitters, probiotics, serotonin

## Abstract

The human gut microbiome has emerged as a crucial regulator of neurophysiological processes by engaging with the central nervous system (CNS) via the microbiota-gut-brain (MGB) axis. One of the most significant ways gut microorganisms influence brain functions is by altering the levels of neurotransmitters. A significant relationship exists between microbial activity and mood, behavior, and cognition. Gut microorganisms can make or break down bioactive substances like serotonin, dopamine, *γ*-aminobutyric acid (GABA), glutamate, acetylcholine, and histamine. These microbial modulations influence precursor availability, receptor sensitivity, synaptic signaling dynamics, and neuroimmune modulation, thereby indirectly shaping neurotransmission within central circuits. These neurochemical effects, particularly involving serotonergic, dopaminergic, GABAergic, and glutamatergic pathways, are mediated through microbial metabolites such as short-chain fatty acids (SCFAs), alterations in tryptophan metabolism, immune system activation, vagal nerve transmission, and the control of the hypothalamic–pituitary–adrenal (HPA) axis. Changes in the composition of the microbiome have been frequently linked to mood disorders, such as depression, anxiety, bipolar disorder, and schizophrenia. The current review integrates findings from preclinical and clinical studies on microbiome-related neurotransmitter modulation, emphasizing novel therapeutics such as probiotics, prebiotics, fecal microbiota transplantation, and dietary alterations. Unlike previous reviews that primarily focus on microbiome composition or therapeutic interventions such as probiotics and fecal microbiota transplantation, this review adopts a neurotransmitter-centered framework, integrating microbial regulation of serotonergic, dopaminergic, GABAergic, glutamatergic, cholinergic, and histaminergic systems with the pathophysiology of mood disorders. Connecting microbiota-driven modulation of neurochemistry to mental outcomes offers a promising adjunctive avenue for mood disorder management, pending rigorous mechanistic and clinical validation.

## Graphical abstract

**Figure:**
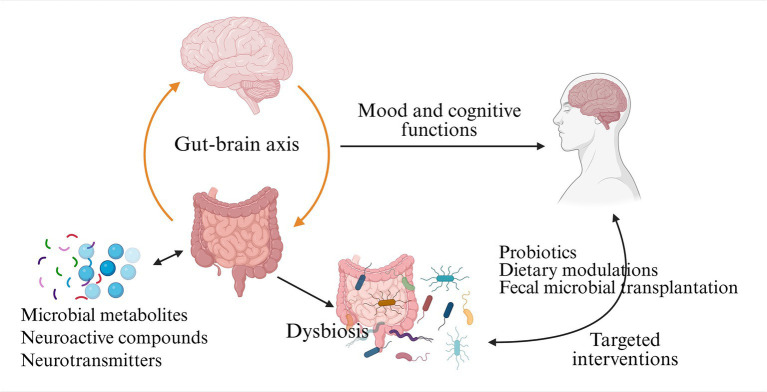
The MGB axis links microbial signals from the intestine to mood and cognition, with dysbiosis modifiable through targeted interventions such as probiotics and dietary modulation.

## 1 Introduction

The human gastrointestinal system hosts a vast and diverse microbial ecosystem, comprising over 100 trillion bacteria from over a thousand species and strains (Liu and Zhu, 2018). The gut microbiome does more than digest food; it also affects metabolism, immunity, and brain function. The microbiota-gut-brain (MGB) axis defines a two-way network that connects gut microbiota, the enteric nervous system (ENS), and the central nervous system (CNS) through neuronal, endocrine, and immunological pathways (Strandwitz, 2018; Mhanna et al., 2024; Barbosa et al., 2025; Dinan et al., 2013; Frost et al., 2014). Dysregulations in the MGB axis correlate with depression, anxiety, autism, schizophrenia, Alzheimer’s disease (AD), and Parkinson’s disease (PD) (Sudo et al., 2004; Bravo et al., 2011; Hsiao et al., 2013; Kelly et al., 2016; Harach et al., 2017; Młynarska et al., 2022; Khaledi et al., 2024; Wei et al., 2024).

Modulating neurotransmitters is a critical process. The gut synthesizes or modulates more than 30 neurotransmitters, such as serotonin, dopamine, *γ*-aminobutyric acid (GABA), norepinephrine, and acetylcholine (Barandouzi et al., 2022; Qu et al., 2024; Luqman et al., 2024; Zhang et al., 2024). More than 90% of serotonin and over half of dopamine come from the gut (Martin et al., 2018; Barandouzi et al., 2022; Alcaino et al., 2025). These chemicals and microbial precursors can enter circulation, modulate vagal signaling, or impact immunological and endocrine functions, influencing brain circuits associated with mood and cognition (Sudo et al., 2004; Yano et al., 2015; Bravo et al., 2011). This link is crucial to major depressive disorder (MDD), which is one of the most common causes of disability and affects about 15% of people over their lifetime (Halvorson et al., 2024). One-third of patients remain unresponsive despite treatment with selective serotonin reuptake inhibitors (SSRIs) and serotonin-norepinephrine reuptake inhibitors (Cuijpers et al., 2024; Tesfamicael et al., 2024). In treatment-resistant depression, renewed attention has been directed toward monoamine oxidase inhibitors, which offer mechanistically broader monoaminergic modulation and may benefit patients unresponsive to first-line agents (Junkes et al., 2025). Dysregulation of the MGB axis, characterized by heightened permeability, dysbiosis, and immunological activation, has been associated with MDD (Mhanna et al., 2024; Jiang et al., 2024; Zhao et al., 2024; Fangous et al., 2019; Wu et al., 2023). Individuals suffering from depression or anxiety frequently exhibit reduced microbial diversity and modifications at the phylum level within Firmicutes-to- Bacteroidetes ratio (Gao et al., 2023; Wu et al., 2023).

Germ-free rodents receiving fecal microbiota from individuals diagnosed with MDD exhibit increased anhedonia, behavioral despair, and altered tryptophan metabolism, providing causal evidence for microbiota-driven modulation of depressive phenotypes (Kelly et al., 2016; Zheng et al., 2016; Sanidad et al., 2024; Zhou et al., 2023). Similarly, transplantation of microbiota associated with prenatal depression induces hippocampal neuroinflammation and depressive-like behaviors in germ-free mice (Cao et al., 2025b). Stress alters microbial composition via the hypothalamic–pituitary–adrenal (HPA) axis, while probiotics alleviate behavioral and biochemical effects (Tan, 2023; Tofani et al., 2025). People with depression have less reelin, an extracellular matrix protein that helps control neuronal development and intestinal barrier integrity; it acts like an antidepressant (Halvorson et al., 2024; Reive et al., 2024). Dysbiosis leads to leaky gut, systemic inflammation, and disruption of the blood–brain barrier (BBB), which causes neuroinflammation in conditions such as depression, schizophrenia, and autism (Huang et al., 2024; Wachamo and Gaultier, 2025). Microbial metabolites and precursors, such as tryptophan and tyrosine, affect the production of serotonin and dopamine. Short-chain fatty acids (SCFAs) control neuroinflammation and synaptic plasticity, linking dysbiosis to AD and PD (Huang et al., 2024; Wachamo and Gaultier, 2025; Liu and Zhu, 2018; Strandwitz, 2018; Chen et al., 2021).

Probiotics, prebiotics, nutrition, and fecal microbiota transplantation (FMT) demonstrate therapeutic potential for mood disorders. Probiotics improve mood, reduce anxiety, and boost cognitive function, whereas FMT provides prolonged benefits for autism (Merkouris et al., 2024; Zheng et al., 2024; Brunocilla et al., 2023; Thangaleela et al., 2022).

In conclusion, gut microbiota influences neurotransmission, mood, and cognition. Disruption of the MGB axis results in psychiatric and neurodegenerative disorders, and restoring microbial homeostasis represents a feasible therapeutic strategy (Karimi et al., 2024; Gao et al., 2025; Wu et al., 2023). Although previous reviews have extensively discussed microbiome-based interventions such as probiotics, prebiotics, and fecal microbiota transplantation, most studies have focused on microbial composition, clinical outcomes, or general gut–brain interactions. Conversely, this review presents a mechanism-focused perspective on neurotransmitters, incorporating microbial effects on serotonin, dopamine, GABA, glutamate, acetylcholine, and histamine, along with neurobiological mechanisms specific to various disorders. This methodology establishes a framework that connects microbial metabolism to neurochemical signaling and psychiatric phenotypes, thus providing a more profound mechanistic understanding and potential pathways for precision psychobiotic treatments. This narrative review, informed by a structured literature search rather than a systematic reviewer. Relevant studies published between January 2000 and March 2025 by searching PubMed, Scopus, Web of Science, and Google Scholar were selected. The searches used combinations of keywords related to the microbiota–gut–brain axis, neurotransmitters, and mood disorders. Studies were selected for their relevance to the impact of the microbiota on neurotransmitter systems and neuropsychiatric effects, with particular emphasis on both mechanistic insights and practical applications. Priority was given to peer-reviewed original research articles, clinical studies, and comprehensive review papers. Additional relevant articles were identified through manual screening of reference lists of key publications. This narrative review aimed to provide a conceptual and integrated summary of important and scientifically relevant literature, rather than a complete or systematic analysis.

## 2 MGB axis: foundations of bidirectional communication

**Figure 1:**
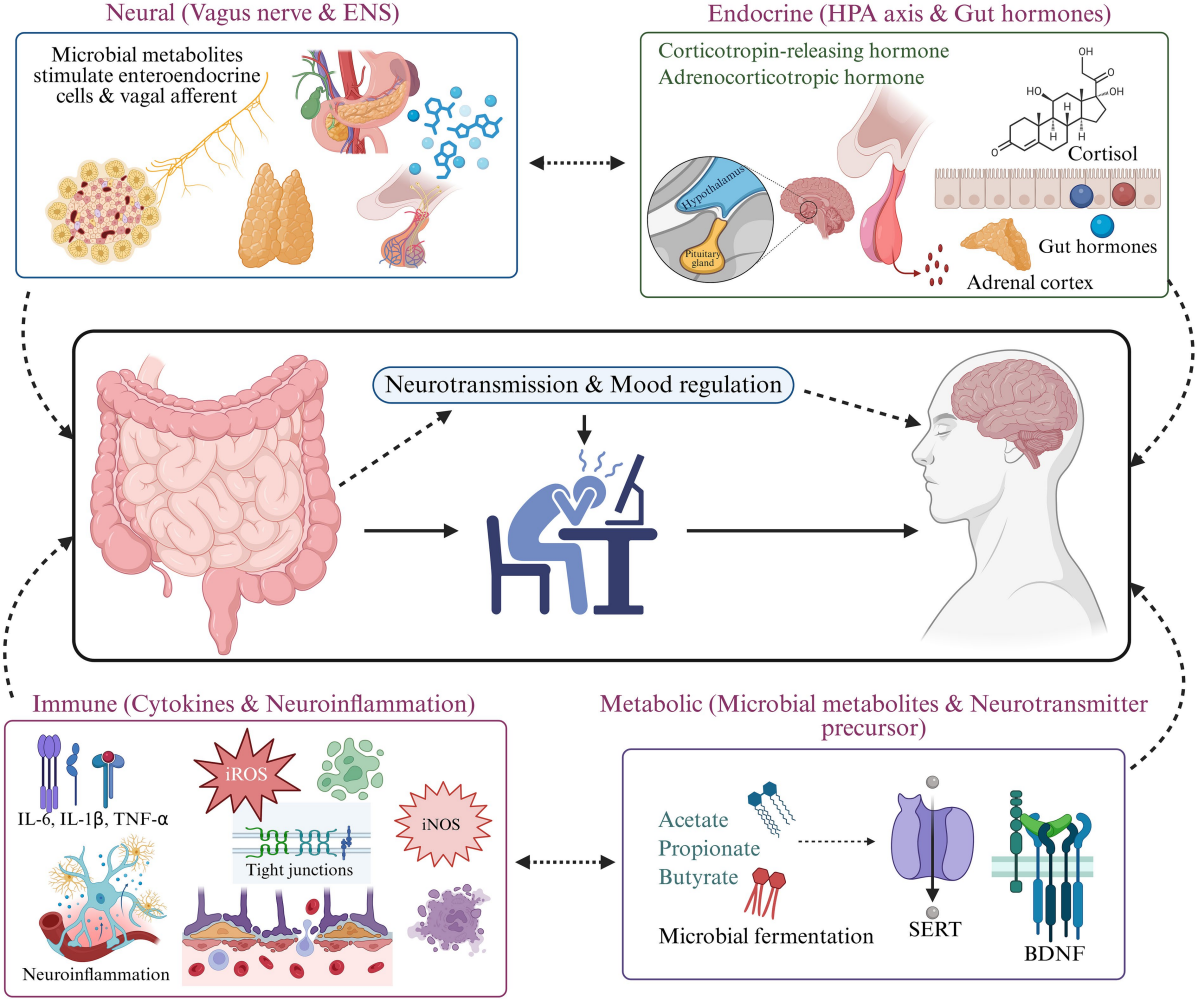
Multidirectional pathways of the microbiota-gut-brain axis influencing neurotransmission and mood regulation. This schematic illustrates the four principal communication routes linking the intestinal microbiome with central nervous system function. Neural signaling occurs via the vagus nerve and enteric nervous system (ENS), where microbial metabolites stimulate enteroendocrine cells and vagal afferents. Endocrine pathways involve activation of the hypothalamic–pituitary–adrenal (HPA) axis and gut hormones, including corticotropin-releasing hormone, adrenocorticotropic hormone, and cortisol. Immune mechanisms reflect cytokine production (e.g., IL-6, IL-1β, TNF-*α*), oxidative and nitrosative stress (iROS, iNOS), disruption of intestinal tight junctions, and neuroinflammation. Metabolic signaling includes microbial fermentation products such as short-chain fatty acids (acetate, propionate, butyrate) and modulation of neurotransmitter-related pathways, including serotonin transporter (SERT) activity and brain-derived neurotrophic factor (BDNF). Together, these interconnected pathways influence neurotransmission, brain function, and mood-related outcomes.

The gut microbiota can send signals to the CNS through several interconnected pathways. Neural signaling is crucial, with the vagus nerve acting as a primary pathway for bidirectional communication between the ENS and the brain, facilitating the influence of microbial metabolites and local neurochemical alterations on central processes (Bravo et al., 2011; Siopi et al., 2023; Jiang et al., 2024; He et al., 2024; Kearns, 2024). Endocrine mechanisms, particularly the HPA axis, exhibit sensitivity to microbial regulation; modifications in gut microbial composition can influence cortisol release and subsequently modify the host stress response (Doenyas et al., 2025; Zhang et al., 2025a). Simultaneously, the immune system represents essential pathways (HPA axis, neural, metabolic pathways) of interaction, as dysbiosis can compromise gut barrier integrity, enhance systemic inflammation, and enable cytokine-mediated alterations that affect neurotransmitter activity and receptor sensitivity (Warren et al., 2024; O'Riordan et al., 2025; Zainal Abidin et al., 2025). At the same time, microbial metabolites, particularly SCFAs, influence host serotonin biosynthesis through induction of tryptophan hydroxylase 1 in enterochromaffin cells (Reigstad et al., 2015; Merlo et al., 2024). Notably, specific gut bacteria possess tyrosine decarboxylase activity capable of converting L-DOPA to dopamine in the intestinal lumen, thereby altering systemic bioavailability of this neurotransmitter (Maini Rekdal et al., 2019). These interconnected pathways highlight the dynamic nature of the MGB axis and show how disturbances in gut microbes can alter neurotransmission and contribute to mood disorders (Liu and Zhu, 2018; Halvorson et al., 2024) (Figure 1).

## 3 Microbiome-mediated regulation of neurotransmitters

### 3.1 Serotonin

Serotonin, 5-hydroxytryptamine (5-HT), is one of the most important neurotransmitters for controlling mood, thought, sleep–wake cycles, and gut motility. Serotonin was once thought to be a central neurotransmitter, but it is now known that more than 90% of it is made in the gastrointestinal system by enterochromaffin cells, and just a small amount is made in the brain itself. The gut microbiota significantly affects this peripheral serotonin pool because it controls the amount of tryptophan available and the activity of critical synthesizing enzymes. For example, spore-forming bacteria can boost the activity of tryptophan hydroxylase 1 (TPH1), the enzyme that controls the rate of serotonin synthesis. This noticeably raises the levels of the neurotransmitter in the mucosa and in the blood (Chen et al., 2021; Li et al., 2024; Alcaino et al., 2025).

The close connection between gut microbiota and the host’s serotonin metabolism goes both ways. Bacterial species, including *Clostridium, Bacillus, Streptococcus, Klebsiella,* and *Escherichia coli*, can synthesize serotonin or serotonin-like substances both *in vitro* and *in vivo*, thereby directly influencing the host’s serotonergic environment. On the other hand, serotonin acts as a signaling molecule that modulates microbial growth, stimulating or inhibiting taxa within the intestinal ecosystem. Serotonin inhibits the growth of *Candida albicans*, but it promotes the development of other species, including *Saccharomyces cerevisiae* and *Enterococcus faecalis*, showing that serotonin has selective effects on microbial community dynamics (Liu et al., 2020; Akram et al., 2023; Xu and Lu, 2025; Ķimse et al., 2024). This demonstrates a reciprocal control in which gut microbiota affects serotonin production, and serotonin, in turn, regulates microbial composition. Bacteria influence host serotonin mainly by altering how tryptophan is metabolized. Under physiological settings, dietary tryptophan can be transformed into serotonin by enterochromaffin cells or redirected to the kynurenine route, the latter frequently linked to neurotoxic metabolites that exacerbate depression and cognitive impairment (Renga et al., 2023; Ramadan et al., 2025). Dysbiosis alters this delicate balance by enhancing kynurenine metabolism at the expense of serotonin synthesis, ultimately decreasing serotonergic tone and predisposing to mood disorders (Xu et al., 2025). Germ-free mice or animals subjected to antibiotic-induced microbiota depletion have modified serotonin levels in both plasma and cerebral areas, alongside increased anxiety-like behaviors, which revert to baseline upon recolonization with specific microbial taxa (Delgado-Ocaña and Cuesta, 2024). These findings underscore a developmental window of opportunity wherein the microbiome influences serotonergic circuitry with enduring effects. The consequences for neuropsychiatric health are significant. Alterations in serotonin signaling have been associated with the pathophysiology of depression, anxiety, and autism spectrum disorder (ASD) (Jiang et al., 2024). Recent meta-analytic evidence further demonstrates that cortical 5-HT2A receptor alterations are consistently associated with major depression and suicidal behavior, underscoring receptor-level serotonergic dysregulation as a critical pathophysiological substrate (Chapman et al., 2025).

Clinical evidence indicates that depressed individuals frequently exhibit diminished plasma tryptophan levels and modified gut microbial composition. Furthermore, fecal microbiota transplantation from depressed donors into germ-free rodents elicits depression-like behaviors, thereby illustrating a causal relationship for microbiome-driven serotonergic dysregulation (Kelly et al., 2016; Faraji et al., 2025). These translational correlations are summarized in Table 1. Similarly, abnormalities in serotonin metabolism have been described in ASD, with altered microbial profiles linked to impaired serotonergic signaling and behavioral symptoms, some of which may be ameliorated by microbiota-targeted interventions (Liu et al., 2019; Xiao et al., 2021; Aziz-Zadeh et al., 2025). Therapeutically, these findings show that the serotonin system is closely linked to the gut and cannot work separately from it. While SSRIs remain the cornerstone of antidepressant treatment, their efficacy appears to be influenced by microbial composition, suggesting that interindividual differences in gut microbiota may partially explain variability in clinical outcomes. Using probiotics like *Lactobacillus plantarum* or *Bifidobacterium infantis*, prebiotics, and fecal transplants are new ways to restore serotonin balance and support mental health (Xu et al., 2023; Xie et al., 2024; Dziedzic et al., 2024; Wang et al., 2023). The microbiome-serotonin axis serves as a key biological bridge between gut health and mental well-being, offering new possibilities for treating mood and neurodevelopmental disorders.

**Table 1:** Microbiome alterations and neurotransmitter disruption across mood and neuropsychiatric disorders.

Disorder	Microbiome alterations	Key neurotransmitter dysregulation	Mechanism	Clinical/Preclinical findings	References
MDD	Reduced α-diversity. Decreased the abundance of *Faecalibacterium* and *Coprococcus*. Increased pro-inflammatory taxa.	Reduced level of serotonin. Altered tryptophan-kynurenine balance. Glutamate imbalance.	Reduced SCFAs production. Increased intestinal permeability. Endotoxin-mediated inflammation. Diversion of tryptophan toward the kynurenine pathway.	FMT from depressed patients induces depressive-like behaviors in rodents. Clinical cohorts show reduced SCFA-producing taxa correlated with symptom severity. Probiotic supplementation demonstrates modest antidepressant effects.	Kelly et al. (2016), Souza et al. (2023), Averina et al. (2024), Mosquera et al. (2024), Rahmannia et al. (2024), and Cao et al. (2025a)
Anxiety disorders	Depletion of *Lactobacillus* and *Bifidobacterium* species	Reduced GABA signaling. Serotonergic instability.	Reduced microbial GABA synthesis. Altered vagal nerve signaling. Hyperactivation of the HPA axis.	Germ-free models exhibit increased anxiety-like behavior reversible with microbial restoration. Probiotic administration improves stress resilience and reduces anxiety symptoms in clinical trials.	Bravo et al. (2011), Siopi et al. (2023), Grau-Del Valle et al. (2023), Rahmannia et al. (2024), and Casertano et al. (2024)
Bipolar disorder	Altered microbial diversity. Immune-associated dysbiosis.	Glutamate-GABA imbalance and dopaminergic instability.	Microbial-driven neuroinflammation. Disruption of excitatory/inhibitory signaling. Metabolic and inflammatory interactions.	Observational studies report immune-associated dysbiosis correlated with mood instability; causal evidence remains limited.	McGuinness et al. (2024), Lin et al. (2024), and Chin Fatt et al. (2023)
Schizophrenia	Dysbiosis with an altered Firmicutes/Bacteroidetes ratio.	Dopamine dysregulation. Glutamatergic dysfunction.	Increased systemic inflammation. Activation of the kynurenine pathway. Microbial modulation of NMDA receptor signaling.	Clinical cohorts show altered microbial composition correlated with cognitive and symptom severity; glutamatergic and dopaminergic disturbances linked to inflammatory signaling.	Ahmed et al. (2024), McGuinness et al. (2024), Okubo et al. (2024), Hanson et al. (2024), and Młynarska et al. (2025)
PD	Increased *Enterococcus faecalis.* Reduced SCFAs producers.	Dopamine depletion. Impaired L-DOPA bioavailability.	Microbial tyrosine decarboxylase activity converting L-DOPA to dopamine in gut. Inflammation-mediated neurodegeneration.	Increased *E. faecalis* associated with reduced L-DOPA bioavailability. Reduced SCFA-producing taxa correlate with neuroinflammation and motor severity.	Maini Rekdal et al. (2019), Menozzi and Schapira (2024), Miyaue et al. (2025), Huang et al. (2024), and Loh et al. (2024)

GABA, Gamma-aminobutyric acid; HPA axis, Hypothalamic–Pituitary–Adrenal axis; L-DOPA, Levodopa (L-3,4-dihydroxyphenylalanine); MDD, Major depressive disorder; NMDA, N-methyl-D-aspartate; PD, Parkinson’s disease; SCFAs, Short-chain fatty acids.

### 3.2 Dopamine

Dopamine, a catecholamine neurotransmitter crucial for reward processing, motivation, and motor control, is recognized to be significantly affected by gut microbiota (Hamamah et al., 2022; Hadrich et al., 2025). Approximately 50% of peripheral dopamine is synthesized within the gastrointestinal tract, primarily by enteric neurons and enteroendocrine cells, although this pool remains largely segregated from central dopaminergic circuits due to BBB constraints (Martin et al., 2018; van Kessel et al., 2019). Some commensal and opportunistic bacteria, like *Escherichia coli, Klebsiella pneumoniae,* and *Morganella morganii*, as well as some strains of *Staphylococcus*, can make dopamine and other catecholamines *in vitro* by decarboxylating l-3,4-dihydroxyphenylalanine (L-DOPA), or similar compounds (Sittipo et al., 2022; Averina et al., 2020; Jabbari Shiadeh et al., 2025). Such findings have provided a biological basis for the concept of microbial endocrinology, whereby microbial communities are not merely passive inhabitants of the gut but active participants in host neurochemical signaling. The influence of gut microbes on dopamine metabolism has been well demonstrated in germ-free animal models. Mice raised in sterile environments show altered dopamine turnover in brain regions such as the striatum and nucleus accumbens, suggesting that the absence of microbial-derived signals disrupts dopaminergic homeostasis (Sittipo et al., 2022; de Wouters d'Oplinter et al., 2022).

Recolonization with defined microbial consortia, particularly *Clostridium* species, restores dopamine and norepinephrine levels in the gut and partially normalizes central dopaminergic signaling (Sittipo et al., 2022). These findings indicate that the microbiota can directly influence catecholamine levels through microbial biosynthesis and indirectly by modulating host enzymes and metabolic pathways (Loh et al., 2024; Xu and Lu, 2025). A striking feature of this relationship is its bidirectionality: dopamine affects the host and acts as a growth and signaling molecule for microbes. Pathogenic strains such as *Escherichia coli* O157:H7 and *Klebsiella pneumoniae* demonstrate enhanced growth, motility, and virulence in the presence of dopamine and norepinephrine, a phenomenon linked to bacterial iron acquisition and quorum sensing (Dowd, 2007; Freestone et al., 2007). Thus, fluctuations in gut dopamine may reshape microbial community dynamics, which in turn feed back into host neurotransmitter balance, establishing a tightly interwoven ecological-neurochemical loop (Strandwitz, 2018; Loh et al., 2024). Disruption of this loop has been associated with several neuropsychiatric and neurodegenerative disorders. Alterations in the gut microbiota are linked to reduced dopaminergic signaling, which contributes to motivational deficits and anhedonia typical of depression, while microbial modulation of dopamine signaling has been associated with schizophrenia and ASD (Ahmed et al., 2024; Abildinova et al., 2024; Młynarska et al., 2025).

Clinically, dopaminergic instability linked to microbial dysbiosis has been associated with motivational deficits in depression, altered reward processing in bipolar disorder, and dopaminergic dysfunction in schizophrenia (Table 1). In PD, dysbiosis not only exacerbates neuroinflammation but also interferes with pharmacological dopamine replacement therapy. Notably, certain gut bacteria, such as *Enterococcus faecalis* possess tyrosine decarboxylase activity that converts L-DOPA into dopamine in the gut lumen, thereby limiting its systemic bioavailability and reducing the efficacy of L-DOPA-based treatment (Maini Rekdal et al., 2019; Menozzi and Schapira, 2024; Takeshige-Amano et al., 2025). This microbial interference has profound implications for disease management, as modulation of the gut microbiota may enhance therapeutic response in PD patients. These findings underscore that dopamine regulation extends beyond classical neuronal pathways and is profoundly shaped by the gut microbiota. By producing, metabolizing, and responding to dopamine, gut bacteria participate in a bidirectional exchange that links microbial ecology to brain function. Therapeutically, this raises the possibility that targeted interventions, such as probiotics, prebiotics, dietary modulation, or inhibition of microbial decarboxylase activity, may provide novel strategies to restore dopaminergic balance in disorders ranging from depression to PD (Ahmed et al., 2024; Młynarska et al., 2025; Miyaue et al., 2025).

### 3.3 GABA

GABA is the principal inhibitory neurotransmitter in the CNS, crucial for maintaining excitatory, inhibitory balance and regulating stress, anxiety, and emotional states (de Leon and Tadi, 2025; Arora et al., 2024). Alterations in GABAergic signaling have been consistently associated with psychiatric disorders such as depression, anxiety, and ASD (Cutler et al., 2023; Johnstone and Cohen Kadosh, 2024). The gut microbiota can modulate GABA levels through both direct biosynthesis and indirect effects on host signaling pathways. Several strains of *Lactobacillus* and *Bifidobacterium* encode glutamate decarboxylase enzymes capable of converting glutamate into GABA, thereby providing an exogenous source of this neurotransmitter (Miri et al., 2023; Ikegami et al., 2024; Liu et al., 2023). Experimental studies using germ-free or antibiotic-treated animals have shown that depletion of the microbiota reduces GABA availability and induces anxiety-like behaviors, whereas colonization with GABA-producing bacteria restores GABA levels and alleviates stress responses (Strandwitz, 2018; Bravo et al., 2011). Notably, *Lactobacillus rhamnosus* JB-1 administration altered GABA receptor expression in distinct brain regions and reduced corticosterone levels, with these effects abolished by vagotomy, highlighting the vagus nerve as a key signaling route in the MGB axis (Siopi et al., 2023). While GABA itself cannot cross the BBB, microbiota-derived GABA influences CNS function through vagal activation, modulation of the HPA axis, immune regulation, and microbial metabolites such as acetate that integrate into hypothalamic GABA metabolism (Belelli et al., 2025). Translational studies further indicate clinical potential, as probiotic supplementation with GABA-producing strains, including *Lactobacillus casei* and *Bifidobacterium*, has been associated with reduced psychological distress and improved mood in humans as shown in Table 1, Casertano et al. (2024), and Braga et al. (2024). Together, these findings establish microbial GABA production as a key mechanism contributing to the anxiolytic and antidepressant effects of specific probiotics, underscoring its therapeutic relevance in neuropsychiatric disorders.

### 3.4 Glutamate

Glutamate, the primary excitatory neurotransmitter, modulates synaptic plasticity and cognitive functions (de León-López et al., 2025). Dysregulated glutamatergic signaling, especially concerning N-methyl-D-aspartate receptor activation, has been associated with depression and schizophrenia (Hanson et al., 2024; Okubo et al., 2024). Recent translational advances indicate that modulation of NMDA and AMPA receptor dynamics underlies the rapid antidepressant effects of glutamatergic agents, further emphasizing glutamate receptor plasticity as a therapeutic target in mood disorders (Freudenberg et al., 2025). Microbial metabolites, such as SCFAs and tryptophan derivatives, can indirectly influence glutamatergic neurotransmission (Takeda et al., 2025; Chen et al., 2025), whereas certain bacteria synthesize glutamate directly (Miri et al., 2023; Zhang et al., 2024). The fragile equilibrium between excitatory glutamate and inhibitory GABA may be affected by microbial makeup, resulting in mood disorders (Gruenbaum et al., 2024; Loh et al., 2024). Longitudinal neuroimaging studies demonstrate that cerebral glutamate trajectories in antipsychotic-naïve first-episode psychosis patients correlate with symptom severity and cognitive outcomes over time, reinforcing the clinical relevance of glutamatergic imbalance in psychotic disorders (Bojesen et al., 2025). This imbalance has been implicated in mood disorders, schizophrenia, and bipolar disorder. In these conditions, inflammatory activation and kynurenine pathway alterations may further disturb NMDA receptor–mediated signaling as shown in Table 1.

### 3.5 Histamine and acetylcholine

Acetylcholine is produced by both the host and specific microorganisms, like *Lactobacillus plantarum*, affecting mood, cognition, and alertness (Chen et al., 2021; He et al., 2024). Microbial acetylcholine synthesis may facilitate cholinergic signaling in the ENS and influence CNS function through vagal pathways (Ortega et al., 2023). Certain bacteria, including *Morganella morganii* and *Klebsiella pneumoniae*, have been implicated in histamine production and neuro-immune signaling, with overactive histaminergic activity potentially exacerbating neuroinflammation and anxiety (Bang et al., 2025). Changes in the microbiome in mood disorders are well documented: in MDD, diminished microbial diversity and lower prevalence of SCFA-producing taxa (*Faecalibacterium prausnitzii*) are observed alongside increased pro-inflammatory bacteria (Grau-Del Valle et al., 2023; Wu et al., 2023). Anxiety disorders correlate with the depletion of *Lactobacillus* and *Bifidobacterium* populations, and probiotic therapy aimed at these strains mitigates anxiety-like behavior (Grau-Del Valle et al., 2023). Dysregulated glutamatergic and GABAergic signaling, resulting from microbial dysbiosis, has been associated with schizophrenia and bipolar disease (Ahmed et al., 2024) (Table 1).

### 3.6 Integrative mechanisms of microbial–neurochemical modulation

Although individual neurotransmitters have been discussed separately, emerging evidence suggests that microbiome–neurochemical interactions operate within an integrated, hierarchical framework rather than through isolated pathways. Three principal mechanistic layers can be distinguished: (1) direct microbial neurotransmitter synthesis, (2) precursor modulation affecting host biosynthetic pathways, and (3) immune–endocrine–metabolic mediation. Direct synthesis of GABA, serotonin analogs, or catecholamines by specific bacterial strains may exert rapid peripheral effects and modulate enteric signaling. However, precursor modulation, particularly of tryptophan and tyrosine, exerts broader systemic effects by altering substrate availability for central neurotransmitter production and shifting metabolic partitioning toward either the serotonergic or the kynurenine pathways. Immune-mediated and metabolic mechanisms appear to have the greatest downstream impact. Microbial metabolites such as SCFAs regulate microglial activation, blood–brain barrier permeability, and synaptic plasticity, while cytokine signaling and HPA axis modulation reshape stress responsivity and neurotransmitter receptor sensitivity (Grundeken and EI Aidy, 2025). Disorder-specific patterns also emerge. MDD is strongly associated with inflammatory signaling and tryptophan–kynurenine imbalance. Anxiety phenotypes demonstrate prominent vagal and GABAergic modulation. PD highlights microbial interference with dopaminergic pharmacokinetics, particularly L-DOPA metabolism. Schizophrenia and bipolar disorder exhibit disturbances in excitatory–inhibitory equilibrium linked to glutamatergic and GABAergic dysregulation. Importantly, environmental exposures during neurodevelopment, including adolescent nicotine exposure, induce persistent neurocircuitry alterations that increase lifelong psychiatric vulnerability, highlighting critical windows during which microbiome–neurotransmitter interactions may exert amplified effects (Reynolds et al., 2025). This mechanistic synthesis highlights synergistic interactions among microbial pathways and shifts the conceptual framework from neurotransmitter-specific effects toward systems-level modulation of neural circuits.

## 4 Translational implications, challenges, and future directions in microbiome-based interventions

The development of microbiome–transmitter interventions has moved from theoretical concepts to clinical trials. Rather than seeing psychobiotics and microbiome modulation as one-size-fits-all solutions, growing evidence suggests that their therapeutic effects are specific to certain disorders and are mechanistically linked to changes in neurotransmitter systems and behavioral symptoms.

Major depressive disorder (MDD)—In cases of MDD, studies consistently report decreased microbial diversity and a loss of SCFA-producing bacteria, along with altered tryptophan–kynurenine metabolism and serotonergic imbalance. Meta-analyses show that probiotic supplements can produce small but consistent antidepressant effects, especially when combined with medication (Mosquera et al., 2024; Rahmannia et al., 2024; Brunocilla et al., 2023). These benefits are thought to involve increased peripheral serotonin, higher SCFA levels, and reduced HPA axis activity (Bertollo et al., 2025). FMT studies support causality: transferring microbiota from depressed individuals can cause despair-like and anhedonic behaviors in rodents (Kelly et al., 2016), and early clinical trials report symptom improvements, though with some methodological limitations (Zhang et al., 2025b). Variability in strains, dosages, and participant features limits reproducibility (Cao et al., 2025a, 2025b).

Anxiety disorders**-**anxiety is often linked to a depletion of *Lactobacillus* and *Bifidobacterium* species and reduced microbial GABA production. Probiotics have shown anxiety-reducing effects in preclinical and clinical studies, likely through modulation of vagal signaling and lowering HPA axis hyperactivity (Rahmannia et al., 2024; Sanidad et al., 2024). Diets rich in prebiotic fibers and fermented foods can promote microbial diversity and SCFA production, possibly stabilizing stress responses and emotional regulation (Schneider et al., 2024; Balasubramanian et al., 2024; Clerici et al., 2025). Short-term probiotic use also improves stress resilience in healthy people, indicating potential preventive benefits (Sarita et al., 2025).

Bipolar disorder and schizophrenia—In bipolar disorder and schizophrenia, microbial imbalance overlaps with glutamatergic–GABAergic dysregulation and dopaminergic instability. While controlled trials are limited, emerging evidence suggests microbiome modulation may reduce inflammation and excitatory–inhibitory imbalance. However, medication, diet, and lifestyle factors complicate findings, and current data are mostly correlational (Chin Fatt et al., 2023).

Precision and mechanism-based strategies—Advances in multi-omics combining metagenomics, metabolomics, and host transcriptomics are starting to reveal how microbes influence neurochemistry. For instance, certain *Clostridium* species promote colonic serotonin production by increasing TPH1 expression (Bai et al., 2024). Biomarkers like plasma serotonin, kynurenine/tryptophan ratios, and neuroimaging can help track target engagement in personalized trials (Hernández-Cacho et al., 2025). The field is shifting from broad probiotics to strain-specific psychobiotics, engineered microbial consortia, and postbiotics aimed at specific neurochemical pathways (Dong and Mayer, 2024; Marano et al., 2025). Combining microbiome therapies with standard drugs might improve outcomes, especially when inflammation or metabolic issues reduce antidepressant effectiveness (Pan et al., 2025).

Challenges and future directions—Despite promising progress, many challenges remain. Proving causality in humans is difficult due to factors such as diet, medication, other health issues, and individual differences in the microbiome (Chin Fatt et al., 2023). Most studies are associative, and standardized methods for strain identification, dosing, FMT procedures, and long-term follow-up are urgently needed (Rosell-Cardona et al., 2025). Additionally, the gut microbiome includes not only bacteria but also archaea, fungi, and viruses, suggesting that single-strain approaches might oversimplify complex interactions (Strandwitz, 2018; Butler et al., 2025). Future research should incorporate ecosystem complexity along with mechanistic insights. Overall, microbiome-based interventions are promising as supplementary strategies rather than immediate replacements in psychiatric care. The goal is to develop personalized, mechanism-based psychobiotics informed by microbial profiles, neurotransmitter measurements, and behavioral assessments, aiming to make microbiota–neurotransmitter modulation a key part of precision psychiatry.

## 5 Conclusion

The gut microbiota has emerged as a dynamic regulator of neurotransmitter systems, reshaping current perspectives on mood and neuropsychiatric disorders. Microbial communities influence serotonergic, dopaminergic, GABAergic, glutamatergic, cholinergic, and histaminergic signaling through integrated neural, immune, endocrine, and metabolic mechanisms. Although accumulating evidence supports the microbiome as a modifiable biological interface linking peripheral physiology with central neurochemistry, translation into clinical psychiatry requires rigorous mechanistic validation. Precision psychobiotic strategies tailored to individual microbial signatures may represent a future adjunct to conventional pharmacotherapy. Continued integration of multi-omics profiling, longitudinal human studies, and biomarker-guided trials will determine the extent to which microbiota-targeted therapies can enhance psychiatric care. The key innovation of this review lies in integrating microbiome research within a unified neurotransmitter-centered framework, providing a mechanistic link between microbial activity and neuropsychiatric outcomes.
